# Erythema nodosum following treatment with dasatinib plus chemotherapy in a patient with myeloid blast phase of chronic myeloid leukemia

**DOI:** 10.1002/ccr3.8223

**Published:** 2023-11-22

**Authors:** Nancy Kassem, Awni Alshurafa, Hesham Elsabah, Halima El Omri

**Affiliations:** ^1^ Pharmacy Department National Center for Cancer Care and Research Hamad Medical Corporation Doha Qatar; ^2^ Department of Hematology National Center for Cancer Care and Research Hamad Medical Corporation Doha Qatar

**Keywords:** chronic myeloid leukemia, cutaneous toxicity, dasatinib, erythema Nodosum

## Abstract

Erythema nodosum (EN) is a type of panniculitis occurring due to various conditions. It can be associated with certain malignancies or manifest as a side effect of drugs. This article presents a unique case of EN in a patient with chronic myeloid leukemia (CML‐blast phase) following dasatinib and chemotherapy. Timely recognition and appropriate management are crucial to alleviate symptoms and consider potential drug‐induced etiology.

## Graphical abstract

Well demarcated erythematous plaques and nodules on the trunk and lower limbs.

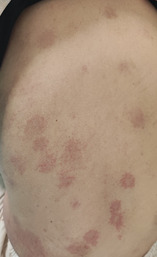

## 1 INTRODUCTION

Erythema nodosum (EN) is a type of septal panniculitis that may occur secondary to a wide variety of conditions (e.g., malignancy, infections, medications, and inflammatory conditions).

It is presented as erythematous, tender, nonulcerated nodules on the bilateral shins and less frequently on the thighs, arms, and neck.^1^

We report a case of erythema nodosum that developed after the initiation of treatment of the myeloid blast phase of chronic myeloid leukemia (CML‐blast phase) in a 40‐year‐old female.

## 2 CASE PRESENTATION

A 40‐year‐old Filipino woman, with no previous medical illness was admitted to our hospital with generalized fatigue and weakness, loss of appetite, weight loss, on/off fever, and left quadrant abdominal pain. Laboratory investigations showed leukocytosis (86.4 × 10^3^/uL) and peripheral smear showed a picture of acute leukemia with morphological features suggestive of transformation from chronic myeloid leukemia. A diagnosis with CML‐blast phase was confirmed, and the patient was started on dasatinib 100 mg, increased to 140 mg after 5 days. On the sixth day, a decision was made to start induction chemotherapy (3 + 7) with intravenous cytarabine (days 1–7) and idarubicin (days 1–3) in addition to dasatinib.

**FIGURE 1:**
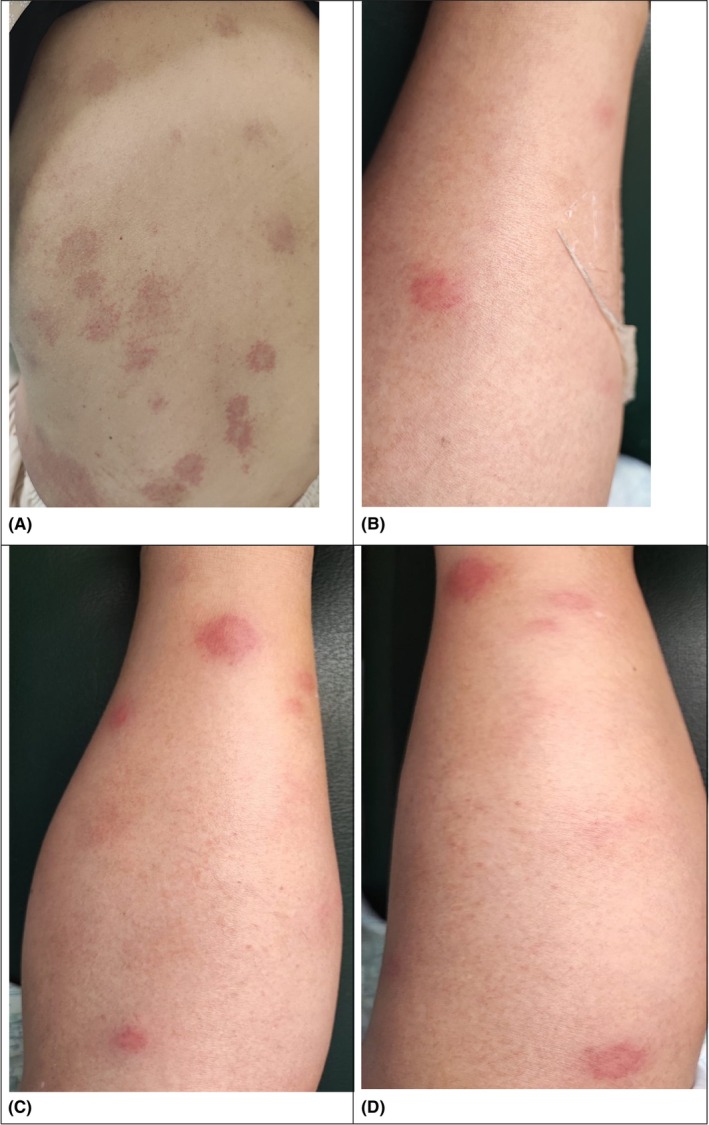
Erythematous and edematous plaques and nodules on trunk (A) and lower limbs (B, C, and D).

Two days after starting chemotherapy, the patient developed high‐grade fever, malaise, and diarrhea; she complained of painful nodules all over her arms, legs, and abdomen (pain score 10/10). She did not have other localized symptoms (no URTI symptoms, no dysuria, and no abdominal pain). Physical examination revealed well‐demarcated erythematous and edematous plaques and nodules on the trunk and lower limbs (Figure 1). They were tender to touch; no oral or genital involvement was noted, and palms/soles were not involved.

Laboratory parameters showed normal WBCs (4.8 × 10^3^/uL), CRP (13.8 mg/L), antistreptolysin O (53 IU/mL), blood, urine, and stool cultures were negative, and viral panel was negative. A 4 mm punch biopsy was taken from the left lower leg skin.

**FIGURE 2:**
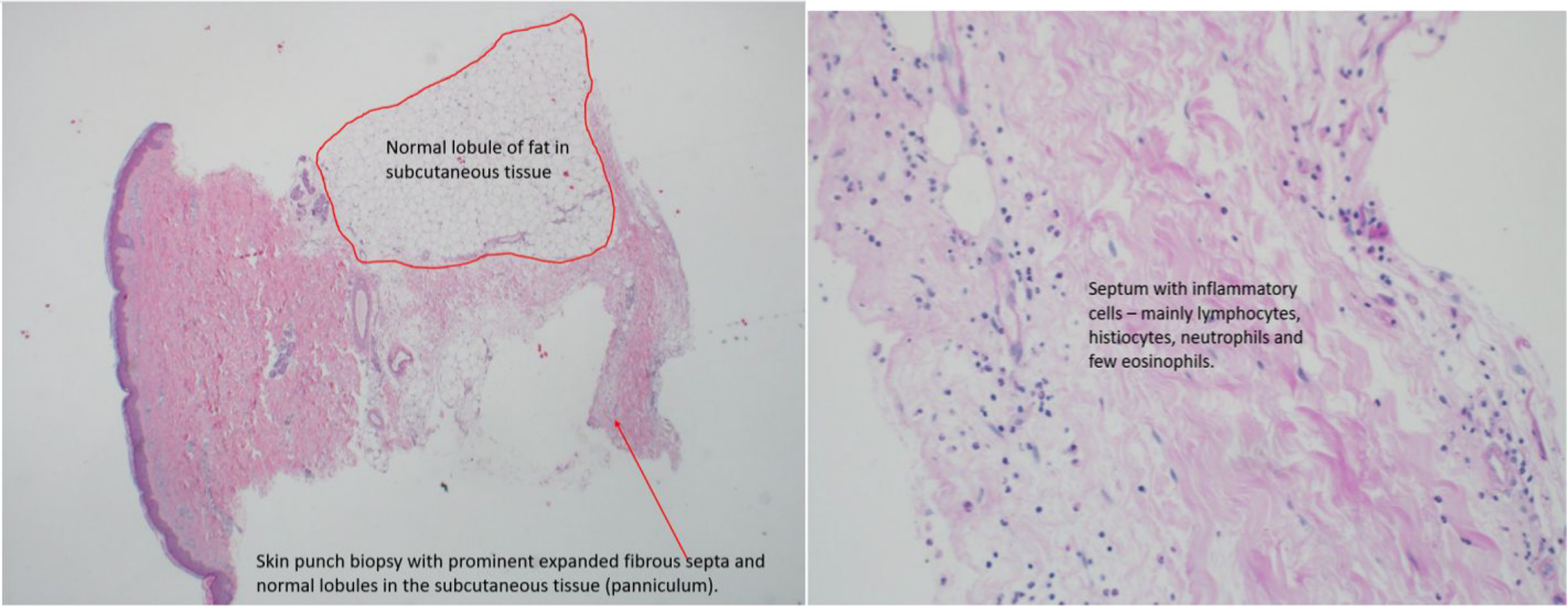
Subcutaneous pathological change with fibrous expansion of septae and mild mixed inflammatory infiltrate (predominantly septal) that includes neutrophils and eosinophils.

Histological examination showed subcutaneous pathological change with fibrous expansion of septae and mild mixed inflammatory infiltrate (predominantly septal) that includes neutrophils and eosinophils. There was no vasculitis. (Figure 2) No microorganism was identified in the biopsy specimen.

A diagnosis of EN was established.

The patient was treated with topical mometasone on the face lesion, clobetasol topical cream on the body's painful red lesions, emollient cream all over the body, and oral prednisolone (20 mg).

Dasatinib dose was reduced to 100 mg daily, idarubicin was already completed, and cytarabine was continued for 7 days then stopped as per protocol.

After 1 week, skin lesions improved, no new lesions appeared, and no pain/tenderness was present; the lesions on the back had resolved with post‐inflammatory hyperpigmentation; other lesions had subsided with a decrease in size and erythema. All lesions were resolved after 2 weeks.

## 3 DISCUSSION

Erythema nodosum is the most frequent form of panniculitis; it is considered a delayed‐type hypersensitivity reaction resulting from exposure to various antigens. It's usually presented as tender round nodules, located mainly on the anterior surface of the lower extremities and however may also affect the thighs, arms, and neck. Cutaneous symptoms may be accompanied or preceded by fever, fatigue, malaise, cough, and arthralgia. EN histologic findings are characterized by features of predominantly septal panniculitis without vasculitis.^1^, ^2^, ^3^

Erythema nodosum can be idiopathic or secondary to multiple causes such as inflammatory conditions, infectious diseases such as viral and rickettsia infections, autoimmune disorders, pregnancy, malignancy, and drugs.^4^

Malignancy‐associated EN is a rare entity; cutaneous nodules can be the first sign of an existent neoplastic disease. Several reports described EN as a presentation of leukemia,^5^, ^6^, ^7^, ^8^, ^9^, ^10^ The exact pathogenesis of EN in leukemia is not well understood, but it is thought to be related to the deposition of immune complexes in the skin.

Erythema nodosum is known to occur with certain drugs such as oral contraceptives, nonsteroidal anti‐inflammatory drugs, and antibiotics. There are many reports documenting associations of chemotherapeutic and biological therapeutic agents with EN such as tumor necrosis factor (TNF) inhibitors, BRAF inhibitors, tyrosine kinase inhibitors, and immune checkpoint modulators.^11^, ^12^, ^13^, ^14^, ^15^, ^16^, ^17^, ^18^, ^19^, ^20^, ^21^, ^22^

Dasatinib is a TKI known to cause several cutaneous toxicities mostly localized and generalized erythema, papular eruptions, and pruritus.^23^ Rare cases of panniculitis have been described in patients treated with dasatinib.^24^, ^25^, ^26^

Although EN is one of the reported uncommon hypersensitivity adverse events in clinical studies and post‐marketing experience of dasatinib^27^; no case reports were found in literature. On the other hand, EN is reported with other TKIs such as imatinib,^17^, ^18^ sorafenib,^19^ nilotinib,^20^ ponatinib,^21^ and other drugs treating leukemia (azacitidine).^28^

Other types of panniculitides are also reported with malignancies and drugs. EN and Sweet syndrome share clinical similarities but can be distinguished by specific features. In our patient's case, the clinical presentation aligns more closely with EN than sweet syndrome. The bilateral tender nodules predominantly localized on the lower extremities, and the absence of mucosal involvement make EN a more likely diagnosis. Additionally, histopathological examination revealed fibrous septal expansion and a mixed inflammatory infiltrate, consistent with septal panniculitis, characteristic of EN. Sweet syndrome, on the other hand, typically presents with neutrophilic infiltration in the dermis without septal panniculitis, and mucosal involvement is more common.^29^ This clinical and histopathological evidence supports the diagnosis of erythema nodosum in our patient.

The cause of EN in our patient could be attributed to leukemia; however, in the majority of cases reported as leukemia presentation, the cutaneous manifestations started before diagnosis of leukemia and subsided after remission. In our patient's case, the symptoms started 2 weeks after diagnosis and resolved before confirmation of remission; this makes it an unlikely probability of being induced by malignancy. On a different note, there was no evidence of infections or autoimmune disorders that might have contributed to the onset of this EN.

The fact that chemotherapeutic agents are associated with several cutaneous toxicities,^30^ and that the nodules appeared after starting 3 + 7 treatment raised the possibility of a correlation between the induction regimen and the development of EN. Dermatological toxicities are not common with idarubicin; in contrast, cytarabine is known to cause skin toxicities including palmar‐plantar erythema, urticaria, maculopapular eruptions, and neutrophilic eccrine hidradenitis.^31^, ^32^, ^33^ Nevertheless, a thorough literature review did not reveal any previous reports of EN in the setting of cytarabine or idarubicin treatment.

On the other hand, the nodules appeared after 1 week of starting dasatinib and 1 day of increasing the dose to 140 mg which suggests that dasatinib is the culprit drug since a temporal relationship between clinical features and the administration of the drug is apparent; yet the possibility of involvement of chemotherapy may still be considered.

## 4 CONCLUSION

We report a unique case of EN occurring in the setting of the CML‐blast phase after treatment with dasatinib plus chemotherapy. This rare cutaneous manifestation may be a potential side effect of dasatinib.

## AUTHOR CONTRIBUTIONS

**Nancy Kassem:** Methodology; writing – original draft; writing – review and editing. **Awni Alshurafa:** Data curation; writing – review and editing. **Hesham El Sabah:** Validation; writing – review and editing. **Halima El Omri:** Validation; writing – review and editing.

## FUNDING INFORMATION

Open access funding provided by the Qatar National Library.

## CONFLICT OF INTEREST STATEMENT

The authors declare that there was conflict no of interest regarding the publication of this case report.

## ETHICS STATEMENT

This study was approved by Hamad Medical Corporation Medical Research Center.

## CONSENT

Written informed consent was obtained from the patient to publish this report in accordance with the journal's patient consent policy.

## Data Availability

Data and materials are available upon reasonable request.
